# Response-retrieval in identity negative priming is modulated by temporal discriminability

**DOI:** 10.3389/fpsyg.2014.00621

**Published:** 2014-06-20

**Authors:** Matthias Mittner, Jörg Behrendt, Uwe Menge, Cora Titz, Marcus Hasselhorn

**Affiliations:** ^1^Department of Psychology, University of TromsøTromsø, Norway; ^2^Georg-Elias-Müller Institute for Psychology, University of GöttingenGöttingen, Germany; ^3^German Institute for International Educational Research (DIPF)Frankfurt am Main, Germany

**Keywords:** negative priming, selective attention, response retrieval, episodic memory, visual attention

## Abstract

Reaction times to previously ignored information are often delayed, a phenomenon referred to as negative priming (NP). Rothermund et al. (2005) proposed that NP is caused by the retrieval of incidental stimulus-response associations when consecutive displays share visual features but require different responses. In two experiments we examined whether the features (color, shape) that reappear in consecutive displays, or their level of processing (early-perceptual, late-semantic) moderate the likelihood that stimulus-response associations are retrieved. Using a perceptual matching task (Experiment 1), NP occurred independently of whether responses were repeated or switched. Only when implementing a semantic-matching task (Experiment 2), negative priming was determined by response-repetition as predicted by response-retrieval theory. The results can be explained in terms of a task-dependent temporal discrimination process (Milliken et al., 1998): Response-relevant features are encoded more strongly and/or are more likely to be retrieved than irrelevant features.

## 1. Introduction

**Figure 1:**
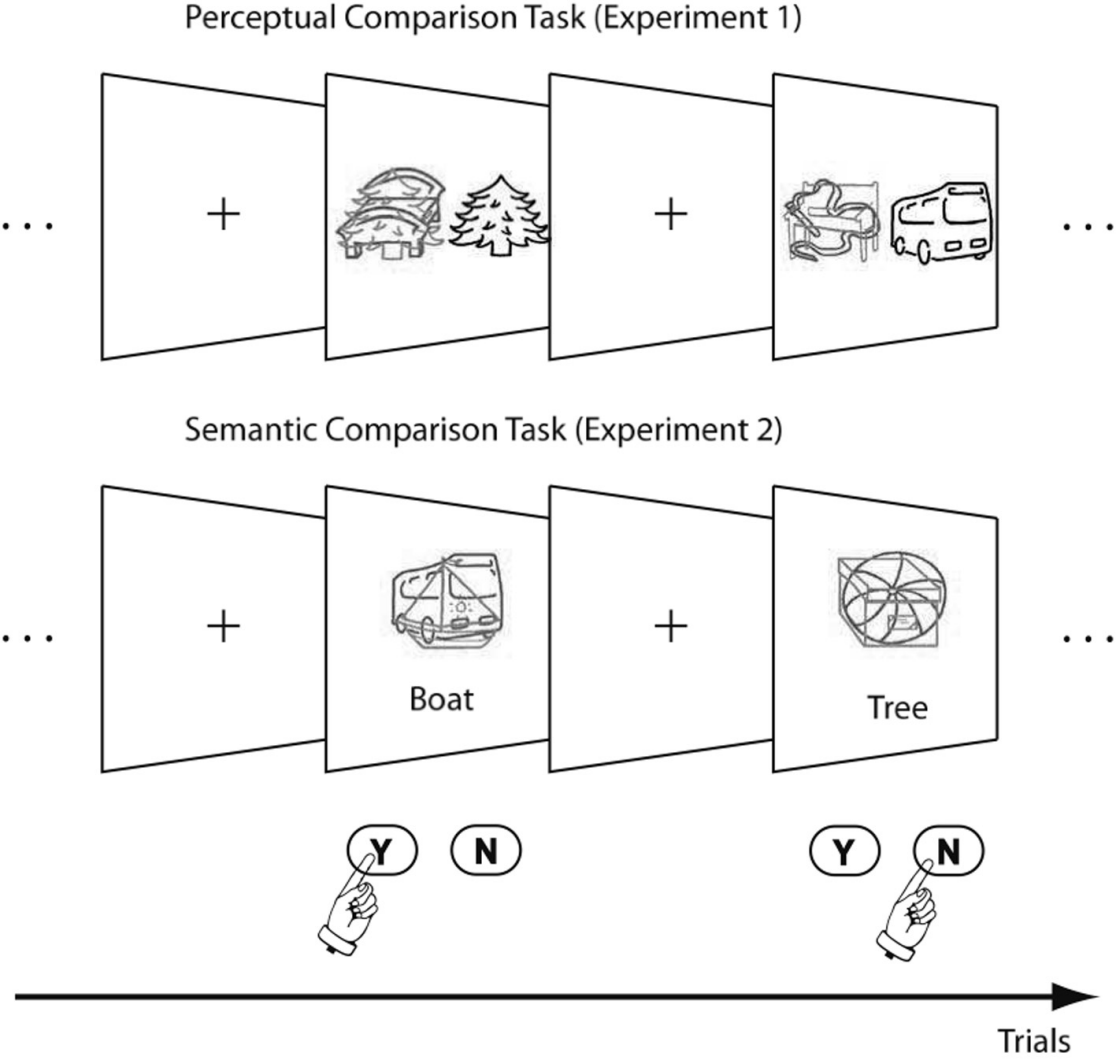
**Example Stimuli for the two experiments**. The green (light-gray) target had to be compared to the gray (black) reference object or word.

The ability to attend to relevant and to ignore irrelevant stimuli is crucial for perceptual-motor functioning. Much of the insight into such processes comes from the study of Negative Priming (NP), which refers to the finding that responses to previously ignored items are usually delayed (Tipper, 1985). More concretely, NP is usually implemented experimentally by presenting two stimuli simultaneously that differ along a response-relevant dimension, e.g., color red vs. green. In the identity version of NP used in the current paper (see Ihrke et al., 2011), subjects have to respond to the identity of the target object identified by a specific color (e.g., green) and to ignore the distractor object of the other color (red). The NP condition is implemented, whenever the stimulus that was ignored in trial *n* − 1 (prime) becomes the target stimulus in trial *n* (probe; see Figure 1).

Early theories of NP focussed exclusively on the NP condition (e.g., inhibition theory; Tipper, 1985). However, the larger set of conditions realized when repeating target or distractor stimuli from prime to probe in different roles (e.g., the positive priming condition when the target stimulus is repeated identically) leads to a broad range of phenomena that theoretical accounts of NP have to cover. More recently, by regarding the NP paradigm as partial repetition of stimulus- and response-features, it has also been used for the investigation of stimulus-response bindings (Rothermund et al., 2005; Mayr et al., 2011; Henson et al., 2014). This perspective justifies the usage of the NP phenomenon in a more general setting to study how selective attention can influence the binding and retrieval of stimulus-response episodes (Henson et al., 2014) or, more specifically, event-files (Hommel, 1998, 2004, 2005).

The approach of using NP for the investigation of event-file binding and -retrieval is particularly attractive because NP is known to be quite general, i.e., it emerges in a variety of experimental tasks, including naming (e.g., Malley and Strayer, 1995), lexical decisions (e.g., Neumann et al., 1999), localization (e.g., Tipper et al., 1990), categorization (e.g., Tipper and Baylis, 1987) and matching (e.g., Rothermund et al., 2005). NP has also been induced by a variety of stimuli, including letters (e.g., Rothermund et al., 2005), words (e.g., Neill, 1997), numbers (e.g., Lammertyn and Fias, 2005), common objects (e.g., Kramer and Strayer, 2001) and non-sense shapes (e.g., Treisman and DeSchepper, 1996). The fact that NP is prominent in many situations justifies the conclusion that it reflects a general characteristic of the human attentional system. In spite of this generality, numerous factors have been identified that can modulate NP. This fact reflects both, a challenge and an opportunity for empirical research—a challenge, because NP can appear to be unstable to minor variations and an opportunity because the analysis of modulating factors can give insights into the underlying mechanisms.

The explanation of NP in terms of binding and retrieval of event-files has its roots in the episodic retrieval theory (e.g., Neill et al., 1992; Neill, 1997). Episodic retrieval theory asserts that NP is essentially a memory phenomenon. The basic idea is that a repeating “similar” situation triggers the retrieval of “relevant” memory traces that may be able to support the processing of the current task. In the case of identity NP case, perceptual similarity is established because the distractor object is repeated in the NP condition (although in a different color). However, because the distractor is equipped with “do-not-respond” information in the memory-trace that has been attached during encoding, the retrieved memory trace conflicts with the currently required task and therefore results in a delay. More recent retrieval-based accounts go a step further by abandoning the assumption of distractor-specific processing, i.e., the binding of do-not-respond information to the distractor. The TIP/TAP theory transfer (in-)appropriate processing, TIP/TAP; Neill and Mathis, 1998; Neill, 2007, e.g., assumes that processing operations that have been carried out during the prime episode are reinstated during probe processing. The response-retrieval theory developed by Rothermund et al. (2005) argues along similar lines but is slightly more specific, i.e., it assumes that the prime response is retrieved in the probe. Both theories assume that an action associated with a stimulus is stored together in an epsidode. The crucial point in this theory is that this binding between stimulus and response is purely incidental, i.e., the response is bound to all stimuli, not only the target or distractor. The response-retrieval theory thereby creates an interesting link to the literature on stimulus-response bindings studied under the label of *event-files* (Hommel, 1998, 2004, 2005; Zmigrod et al., 2009).

In this line of research, the formation of perception-action bindings are studied by orthogonally varying perceptual feature- and response-repetitions. An interaction between these two factors is seen as evidence for the successful binding and retrieval of stimulus-response associations. The reasoning underlying this argument is based on the early work on feature-feature bindings (object-files; Kahneman et al., 1992) and is as follows. When the stimuli in two displays are similar (i.e., when they share perceptual features), a binding formed in the first display is retrieved during processing of the second. In the case of event-files, this binding is assumed to contain both perceptual and action features, i.e., also the response. Therefore, if the response required in the second display is identical to the retrieved one, the processing may be facilitated while it should be delayed when the required and retrieved responses mismatch.

This line of argument can be readily transferred to the NP paradigm. Repetition of the distractor in the probe triggers retrieval of the prime event-file including the prime response which delays the responding in the case of a mismatch. This perspective highlights a critical confound that has been present in many empirical studies on identity NP so far: When identity is to be responded to, NP trials always require a response-change from prime to probe. Rothermund et al. (2005) argue that the NP effect would disappear or reverse when NP trials are realized without requiring a response-switch. The authors were able to show that NP in a letter matching task depends on whether the required response changes from the first to the second appearance of a stimulus (Rothermund et al., 2005, experiment 4) and this finding has been found to extend to many experimental conditions, e.g., various sorts of letter matching tasks (Frings et al., 2007), auditory categorization (Mayr et al., 2011) and picture-word comparison (Ihrke et al., 2011).

It is obvious that the argument that stimulus- and response-repetitions should interact extends beyond the NP condition. In fact, as far as response-retrieval theory is concerned, *any* repetition from prime to probe should induce retrieval and hence result in the stimulus- × response-repetition interaction. This point has been investigated in a recent study by Ihrke et al. (2011) which implemented all four single-stimulus repetition conditions that are possible in this setup: Identical repetitions include the target-repetition (TT) and the distractor-repetition (DD) condition while the distractor-to-target (DT) and target-to-distractor (TD) condition are partial repetitions because an identical stimulus is repeated in a different color (this terminology is borrowed from Christie and Klein, 2001). Ihrke et al. (2011) report that rather than being independent of priming condition, response-retrieval is modulated by attention: Only stimulus-repetitions where the repeated stimulus was attended in the probe resulted in the stimulus- × response-repetition interaction that indicates that the retrieved response conflicted with the currently required one. This result, congruent with evidence presented by Moeller and Frings (2014), shows that stimulus-response bindings and retrieval are not merely incidental but that these processes are modulated by attention.

Along the same lines, the current study investigates the boundary conditions posed by attention under which response-retrieval operates. While Ihrke et al. (2011) manipulated the attention to either the distractor or target stimulus in either prime or probe, the current study directs the attention to specific stimulus features by manipulating the depth-of-processing required by the experimental task. More specifically, we investigate whether the retrieval of stimulus-response bindings is dependent on whether a perceptual or a semantic matching task is used. This research is motivated by conflicting results in two NP studies. While Ihrke et al. (2011) found the discussed stimulus- × response-repetition interaction, an earlier study using a perceptual comparison task for analyzing age-related specificities of NP by Kramer and Strayer (2001) did not report this interaction. To be more specific, we have to analyze the setup used by the two studies (see Figure 1). Both studies implemented a matching task where three stimuli were presented simultaneously: (1) a target line drawing, which was superimposed by (2) a distractor line drawing in a different color and (3) a reference stimulus. The experimental task was to compare the target to the reference word and to respond with “match” or “mismatch”, accordingly. While the study by Kramer and Strayer (2001) used a perceptual matching task (i.e., the reference was a line-drawing in a neutral color), a semantic matching task was implemented by Ihrke et al. (2011), i.e., the reference stimulus was a word.

From these experimental results, we derive the hypothesis that a processing of the prime stimuli on a semantic or phonetic level might be a necessary prerequisite for the response-retrieval to occur. The idea is that the prime memory trace contains merely perceptual features in the one case and a semantic representation of the stimuli in the other. This assumption is in accordance with the empirical studies referred to above that found support for response-retrieval: The stimuli were processed on a semantic level because letter-reading is sufficiently overlearned to result in an automated internal vocalization (Rothermund et al., 2005; Frings et al., 2007) and comparing a pictogram to a word requires a semantic representation of the objects (Ihrke et al., 2011).

A theoretical rationale for the hypothesis that the depth of processing (i.e., whether the stimuli have been processed on a perceptual or a semantic level) can influence the content of the memory trace and response-retrieval, is based on the temporal discrimination theory by Milliken et al. (1998). These authors argue similar to Logan (1988) that retrieval can possibly support the response to previously seen items but that it depends on an initial evaluation of the display whether retrieval is actually carried out. In their model, an early perceptual scanning (or orienting) process determines whether a sufficient similarity of current situation and a memory trace is present which increases the probability that retrieval can help to resolve the current task. In case of no repeating stimuli, retrieval is unnecessary and the response has to be developed by direct processing of the stimuli. A key prediction of this theory is that a partial match between the episodes can actually lead to poorer performance than no match at all because the scanning process needs more time to confirm dissimilarity.

We argue that the perceptual scanning process is more likely to perceive the dissimilarity in color between prime distractor and probe target in the case of the perceptual comparison task, because it favors the creation of memory traces containing perceptual features and because the retrieval is biased in favor of the focused features. Therefore, the scanning process would classify the DT condition as a mismatch and would not initiate a response retrieval leading to the missing interaction in Kramer and Strayer's (2001) study. On the other hand, the emphasis on stimulus identity rather than perceptual appearance in the semantic comparison task implemented in Ihrke et al.'s (2011) study could have triggered the scanning process to consider stimuli that are of a different color but same identity to be sufficiently similar to initiate retrieval thereby leading to the observed interaction.

We put this hypothesis to a test by realizing two experiments that replicate Kramer and Strayer (2001) and Ihrke et al.'s (2011) studies in a common experimental setting. Both experiments differ in how the target is compared to the reference stimulus: While Experiment 1 requires to resolve only perceptual identity (the target has to be compared to an object), Experiment 2 aims at investigating effects from semantic comparison (the target has to be compared to a written word; see Figure 1). It is necessary to conduct both experiments together, since NP effects are known to be affected by subtle experimental manipulations, such as RSI (e.g., Neill and Valdes, 1992), composition of the trial sequence (e.g., Kane et al., 1997) or distractor saliency (Lavie and Fox, 2000; Grison and Strayer, 2001). Even though the studies by Kramer and Strayer (2001) and Ihrke et al. (2011) appear to be very similar at first sight, there are a number of differences that could have influenced the priming effects (see introduction to Experiment 1) and implementing both experiments within the same framework is therefore necessary, especially in the light of a direct statistical comparison.

We follow the typical approach of experimental studies of the negative priming effect that usually implement a contrast between the positive priming or attended repetition (TT) and the NP or ignored repetition (DT) condition. Ihrke et al. (2011) have shown that response-retrieval depends on the relevance of the repeated stimulus, i.e., only conditions in which the probe target has been seen in the prime elicit response-retrieval—obviously, the negative (DT) and positive priming (TT) condition satisfy this constraint. In addition, we included a reversed repetition condition (Schrobsdorff et al., 2007; Titz et al., 2008) in which both target and distractor reappeared but in reversed roles (distractor-to-target and target-to-distractor, DTTD). Within the theoretical framework by Milliken et al. (1998), reversed repetition is an interesting condition because it fits nicely into the match-mismatch argument: While TT is obviously a condition with a good perceptual match for the scanning process, DT has intermediate similarity (because one stimulus changes color) and control trials have poor similarity. The reversed repetition (DTTD) condition is on the one hand the most similar condition, because both stimuli reappear in the probe, but on the other hand a very dissimilar one, because both objects change color. If our argument for the dependence of retrieval on task in the two experiments is correct, the DTTD condition should produce no response-repetition × priming interaction in the perceptual comparison task realized in Experiment 1 (because of the obvious dissimilarity of red/green stimuli) and a significant priming × response-repetition interaction in the semantic comparison task (Experiment 2).

## 2. Experiment 1

Experiment 1 implemented a design that is similar to the one by Kramer and Strayer (2001). Overlapping target and distractor pictograms had to be compared to a reference pictogram. We expected to replicate the finding of Kramer and Strayer that for partial repetitions, no interaction between priming and response-repetition is present: The perceptual scanning process proposed by Milliken et al. (1998) should not initiate the retrieval of the prime response. When taking a closer look at the experiment by Kramer and Strayer (2001), there are important deviations from Ihrke et al.'s (2011) study apart from the intended manipulation of the reference stimulus that could offer alternative explanations for the diverging results in the two studies.

First, and most importantly, while Ihrke et al. used a small set of only 5 different stimuli, Kramer and Strayer used a comprehensive array of different stimuli because they intended to investigate stimulus repetition effects on NP. The size of the stimulus set can have strong influences on the priming effects, where NP is usually most robust for small stimuli sets (Malley and Strayer, 1995). Strayer and Grison (1999) found that NP increased with increasing stimulus familiarity, while DeSchepper and Treisman (1996) found the opposite result. The conflict was resolved by Nagai and Yokosawa (2003) who argue that the mixing or simultaneous presentation of familiar and unfamiliar stimuli was responsible for the opposing results. It is unclear however, how such effects could influence the response-retrieval specific interaction we are looking for and it is therefore necessary to replicate Kramer and Strayer's (2001) result with a small stimulus set.

Furthermore, the relative number of priming and control trials was different in the two studies. While Kramer and Strayer used a balanced number of control and priming trials (i.e., twice as many controls as both DT and TT trials), Ihrke et al. employed a rather strong over-representation of prime trials (four times as many priming trials). The resulting bias for repeating objects might have led the subjects to strategically favor retrieval-based processing and this could have caused the response-repetition × priming interaction to be present in Ihrke et al. (2011) but not Kramer and Strayer's (2001) study. That the subject's strategic set may influence NP effects is a well-known fact since Lowe (1979) and Moore's (1994) studies in which they showed that NP disappeared for single-trial probes when they were interleaved with target/distractor probes but not for single-trial probes alone. This attentional set was also a prominent feature of Tipper and Cranston's (1985) and Milliken et al.'s (1998) theoretical work. The different number of stimulus-repetitions might therefore very well have produced the observed difference between Kramer and Strayer (2001) and Ihrke et al.'s (2011) study.

Furthermore, one aspect of Kramer and Strayer's (2001) study is not consistent with the temporal discrimination account by Milliken et al. (1998): The theory would have expected a significant interaction between positive priming (TT) and response-repetition. This prediction results from the consideration, that the perceptual identity of prime and probe target should have caused the perceptual scanning process to initiate the response retrieval. Therefore, while the retrieved response should have resulted in a conflict in response-switch trials (delaying the probe response), reaction times in response repetition trials should have been accelerated by the match of retrieved and required responses. It is possible that some aspect of Kramer and Strayer's (2001) study prevented the interaction to occur. While it doesn't make sense to speculate about such factors here, a failure to find this interaction for the TT condition in our experiment would falsify our hypothesis, that response-retrieval is modulated by an early, perceptual scanning process.

In summary, we expect the interaction between priming and response-repetition to be present for the target repetition (TT) but insignificant for the DT and the DTTD condition, due to the perceptual comparison required by the task.

### 2.1. Methods

#### 2.1.1. Participants and design

Participants were 24 adults (undergraduate students from the University of Göttingen, Germany: 5 male, 19 female; mean age 23.3 years, *SD* = 3.5). All participants received course credit or were paid 8 EUR (≈ 10 USD) for taking part in the study. Effects of priming condition and response repetition were studied within a 4 (priming conditions: CO, DT, TT, DTTD) × 2 (response repetition: same vs. different) within subject design.

#### 2.1.2. Materials and apparatus

Ten line-drawings of familiar objects were plotted in green, red and gray color (rgb coordinates were {0, 255, 0} for green images; {255, 0, 0} for red ones; {200, 200, 200} for the gray images). The following objects were depicted: ball, tree, bench, book, boat, bus, box, beard, bed, and ribbon. The tasks required perceptual matching as in the procedure used by Kramer and Strayer (2001), see Figure 1 (top).

Participants had to process a continuous series of displays where display *n* served as prime for display *n* + 1 and as probe for display *n* − 1. The response-to-stimulus interval (RSI) was set to 500 ms. Each participant had to process a total of 430 displays, and additionally 126 practice trials. The display sequences were presented in 10 blocks of 43 trials each (the first three displays of each block were not analyzed due to possible transient effects). Each display contained (1) a green stimulus as the target (2) a red stimulus as the distractor and (3) a gray stimulus for reference. The target superimposed the distractor by 80% and objects were located to the left of the midpoint of the screen. The gray reference stimulus was presented to the right of the midpoint. Responses depended on whether the target matched the reference stimulus (“yes/no”) and were given by key-presses on a standard keyboard (left hand: “ctrl”-key, right hand: “enter”-key on the numeric keypad). All line drawings appeared an equal number of times as target, distractor or reference stimulus per prime condition. Assignment of keys to responses was balanced across subjects.

For one half of the trials the reference stimulus corresponded to the target, for the other half it did not. The reference stimulus never corresponded to the distracting object and was unrelated in both the prime and probe. A quarter of the prime probe couples were unrelated control trials (CO). Another 25% of the trials, the prime distractor repeated as target in the probe display DT). In another 25%, not only the prime distractor reoccurred as probe target but the target from the prime also reoccurred as the distractor in the probe (DTTD). The remaining trials were a mixture of single target trials (ST) and trials in which the prime target reoccurred as the probe target (positive priming, TT). ST trials were included for reasons irrelevant to the current studies and will not be analyzed^1^. Trials were presented in an unpredictable (pseudo-randomized) order that was optimized to avoid trial-sequence structure (Ihrke and Behrendt, 2011). The response relationship was varied by requiring that either the same or a different response key for the prime and probe responses had to be pressed (response-repetition, “yes-yes” or “no-no” vs. response-switch, “yes-no” or “no-yes”). Responses, response switches, and response repetitions were balanced across priming conditions.

#### 2.1.3. Procedure

Participants were told that they had to decide whether a drawing with green lines that superimposed a drawing in red lines matched a gray pictogram while ignoring the red drawing. To record their decision, participants pressed the corresponding “yes” or “no” button on the keyboard. There was a short, self-paced break after each block of 43 trials. After subjects had completed the first half of the experiment, they completed a vocabulary test (Schmidt and Metzler, 1992) and a short fluid intelligence test (Digit Symbol Substitution Test, Wechsler, 1958). Finally, the experimenter prompted the participants for any difficulties regarding the experiment, e.g., in identifying the objects.

### 2.2. Results

Trials in which an error occurred as well as trials directly following an error trial (3.0%) were not further analyzed. Trials with response latencies of less than 250 ms or which were more than two standard deviations above the individual means for each participant and condition were excluded as outliers (4.6%). Aggregated mean reaction times, standard deviations, and error percentages are reported in Table 1.

**Table 1:** **Summary of reaction times (RT) and error rates (ER) for Experiment 1**.

	**Mean reaction time/Error rates^a^^b^**				**RRE^c^**	
	**Different response**		**Same response**			
	**RT**	**ER**	**RT**	**ER**	**RT**	**ER**
Control	607.4 (66.0)	3.33 (3.5)	623.8 (60.3)	3.25 (3.1)	16.4	−0.08
DT (NP)	621.3 (66.6)	2.78 (3.1)	634.8 (71.9)	4.17 (3.3)	13.5	1.39
DTTD	624.4 (67.4)	3.06 (3.1)	633.7 (68.7)	3.51 (4.4)	9.3	0.45
TT (PP)	625.3 (75.7)	4.33 (4.1)	603.4 (86.4)	0.50 (1.4)	−21.9	−3.83
	**Priming effects^d^**					
DT (NP)	−13.9	0.56	−11.1	−0.92		
DTTD	−17.0	0.27	−9.9	−0.26		
TT (PP)	−17.9	−1.00	20.3	2.75		

a *Percentage of wrong response per condition*.

b *Standard-deviation in parentheses*.

c *Response-Repetition Effect, same-different*.

d *Difference of control and priming condition*.

**Figure 2:**
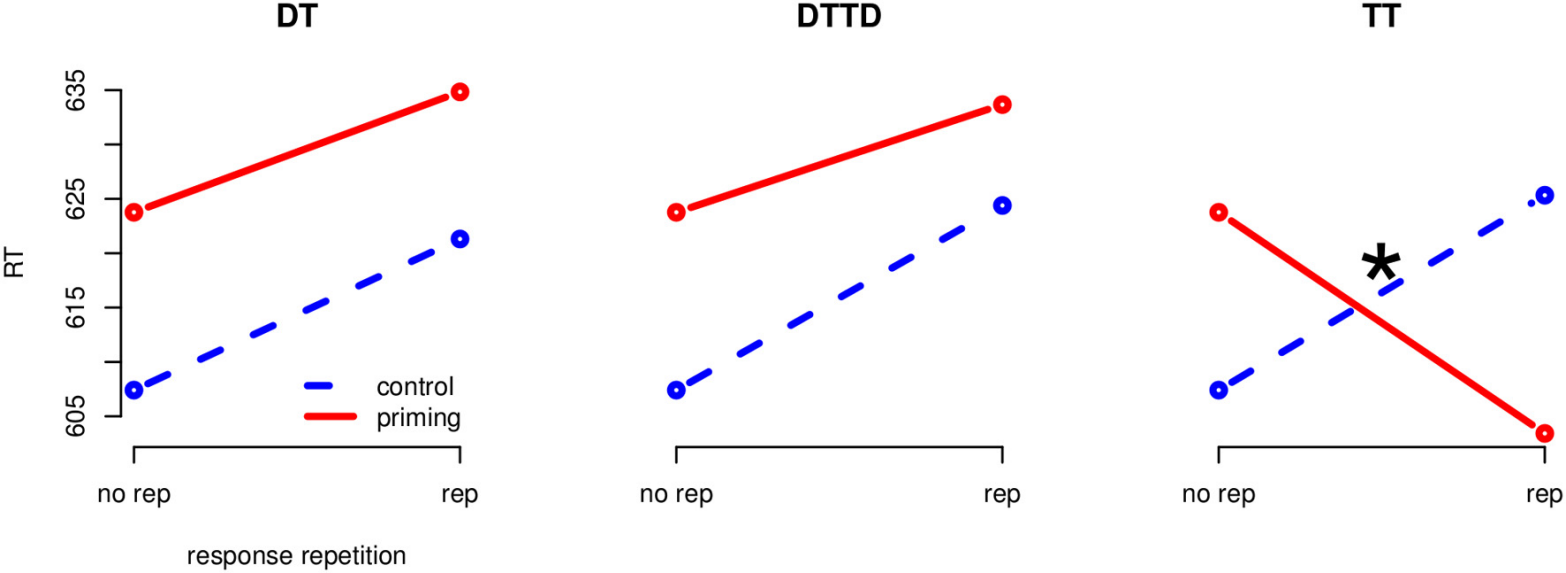
**Separate interaction plots for all priming conditions (Experiment 1)**. Blue, stippled line is the control condition, red solid line the corresponding priming condition. The “*” indicates significance at the 0.05 level.

The global 4 (priming: CO, DT, TT, DTTD) × 2 (response-repetition: same, different) analysis of variance (ANOVA) on reaction times, treating the factors as repeated measures, revealed an interaction of priming and response-repetition *F*_(3, 69)_ = 17.22, *MSE* = 218.83, *p* < 0.001, η^2^_*G*_ = 0.012^2^. There were no main effects of priming or response-relation. To analyze the effects for each of the three stimulus repetition conditions (TT, DT, and DTTD), we applied separate 2 (Priming: control vs. priming) x 2 (Response-repetition) ANOVAs per priming condition which were adjusted adjusted for multiple testing using Holm's (1979) method (Figure 2).

#### 2.2.1. (1) Target repetition (TT)

The 2 (CO vs. TT) × 2 (same vs. different) ANOVA revealed an interaction between priming condition and response repetition, *F*_(1, 23)_ = 32.63, *MSE* = 269.21, *p* < 0.001, η^2^_*G*_ = 0.018. There were no main effects. Target repetition priming had a tendency for a significant facilitative effect of 20.3 ms in the response repetition condition, *t*_(23)_ = 1.66, *p* = 0.06, *d* = 0.27 but delayed the reaction time by 17.9 ms whenever responses in prime and probe were different, *t*_(23)_ = −1.77, *p* < 0.05, *d* = 0.25.

#### 2.2.2. (2) Distractor to target (DT)

A 2 (CO vs. DT) × 2 (same vs. different) ANOVA revealed a main effect “priming condition”, *F*_(1, 23)_ = 11.33, *MSE* = 330.15, *p* < 0.01, η^2^_*G*_ = 0.010 and a main effect of response repetition, *F*_(1, 23)_ = 15.09, *MSE* = 355.10, *p* < 0.01, η^2^_*G*_ = 0.013. The interaction was not significant, *F* < 1. Distractor to target priming resulted in a delay of 12.5 ms regardless of response repetition, *t*_(47)_ = −4.01, *p* < .01, *d* = 0.19. Response repetitions did, however, have a general delay effect of 15.0 ms, *t*_(47)_ = −4.68, *p* < 0.01, *d* = 0.23, on reaction times.

#### 2.2.3. (3) Distractor to target and target to distractor (DTTD)

The 2 (CO vs. DTTD) × 2 (same vs. different) ANOVA revealed a main effect of priming condition, *F*_(1, 23)_ = 17.29, *MSE* = 250.89, *p* < 0.01, η^2^_*G*_ = 0.011 and a main effect of response repetition, *F*_(1, 23)_ = 10.11, *MSE* = 390.12, *p* < 0.01, η^2^_*G*_ = 0.010. The interaction was not significant. DTTD-priming caused a delay of 13.4 ms regardless of the response repetition, *t*_(47)_ = −4.50, *p* < 0.01, *d* = 0.21. Response repetitions led to a delay of 12.8 ms, *t*_(47)_ = −3.74, *p* < 0.01, *d* = 0.20 as compared to response switches.

#### 2.2.4. Error rates

The corresponding analyses for the error rates are largely consistent with the results from the analyses of the reaction times: There was a significant interaction for TT in the 2 (CO vs. TT) × 2 (response-repetition) ANOVA, *F*_(1, 23)_ = 10.99, *MSE* = 7.68, *p* < 0.01, η^2^_*G*_ = 0.084 but no interaction in the corresponding analyses for DT and DTTD, both *F*'s < 1. All other effects failed to reach significance, which we attribute to a lack of power due to too few errors.

### 2.3. Discussion

The results of Experiment 1 support the predictions derived from our hypothesis that a perceptual orienting process could modulate response-retrieval in a perceptual matching task. We observed a main effect of priming for the conditions in which the distractor was repeated in the probe (DT and DTTD) but no interaction with response-repetition. On the other hand, there was no main effect of priming for the positive priming condition (TT) but a significant priming × response-relation interaction: A combination of target repetition and response repetition led to accelerated reaction times, whereas delayed reaction times were observed when the target repetition was accompanied by a response switch. We could thus show that response-retrieval, in the case of a perceptual comparison task, was only conditionally initiated depending on the characteristics of the repeating stimulus: Only, when the repeated stimulus was perceptually identical (i.e., identical in color and shape), response retrieval was initiated.

The missing interaction for DT and DTTD is in conflict to a strict reading of the response-retrieval theory (Rothermund et al., 2005), which would assume that every prime stimulus should be associated with the prime response and thus, the repetition of any stimulus which has been processed during the prime trial should be able to cause a retrieval of the prime response. By including a flexible discriminative orienting process as proposed by Milliken et al. (1998) however, it is possible to give a satisfactory explanation for the absence of the interaction for DT and DTTD but its presence in the TT condition: Because of the perceptual identity in the target repetition condition, the process was able to retrieve the prime response, thereby producing the priming × response-repetition interaction. In the other two conditions the shape of the repeated stimuli was identical but the color was changed. This intermediate similarity led to a time-consuming orienting process manifested in the main effect of priming for the DT and DTTD conditions. Because the orienting process did finally see the differences between the percepts, no response-retrieval took place as observed by the missing interaction between priming and response-repetition.

In Experiment 1, participants had to decide whether or not the green target line drawing matched the gray standard stimulus. For efficient performance it was not necessary to create more complex semantic phonetic representations of the processed stimuli but comparison could operate on a purely perceptual level. Responses were therefore associated with relevant perceptual aspects of the stimuli. As a result a stimulus repetition lead to response retrieval when the stimulus was repeated with all of its relevant perceptual features but not in the case of a partial repetition. This was only the case for TT-trials. In the DT and DTTD-trials the repeated stimuli differed in their response relevant perceptual dimension making retrieval unlikely. Still, the stimuli were similar enough to cause the attentional system to spend some time on the orienting process (perceptual scanning), leading to the observed main effect of priming (Milliken et al., 1998).

We also observed a main effect of response-repetition in the control, DT and the DTTD conditions, indicating that reaction times were prolonged when the response had to be repeated but the stimuli changed. This finding, known as response-repetition effect (RRE), is a common phenomenon in two-alternative-forced-choice (2-AFC) tasks which are characterized by only a few alternative responses for various stimuli (Smith, 1968; Neill et al., 1994; Kleinsorge, 1999; Marczinski et al., 2003). Marczinski et al. (2003) argue that not only does the repetition of a previously processed stimulus lead to the retrieval of the associated response, but the response repetition itself also represents a possible source of interference. During the processing of the prime display, the response is associated with specific stimuli. If the same response has to be given immediately afterwards, this association still prevails, thus accelerating reaction times as long as some of these stimuli are also repeated. In cases where stimuli are not repeated the partial overlap in the response requirement can result in interference. The authors argue that it could be difficult to link an action to a percept when it has just recently been linked to another percept. This would lead to delayed reaction times in trials in which the previous response has to be given again for a completely new percept, i.e., in control trials. However, the finding that the RRE is present also in the DT and DTTD condition and that it is statistically indistinguishable from the control condition supports our conclusion that the stimuli were classified as new by the cognitive system: The response-repetition effect behave in much the same way as in the control condition, indicating that a mismatch between percepts and responses has been noted.

Despite the methodical differences discussed above, the results from Experiment 1 are largely in agreement with those obtained by Kramer and Strayer (2001): We did not find an interaction between response-relation and priming in general and there was a main effect of priming. However, we observed the response-repetition × priming interaction for the target-repetition condition whereas Kramer and Strayer did not. This difference is not easily explained but it could have been caused by the subject's strategic set: Kramer and Strayer (2001) varied the number of times each object had been seen over time and found that their NP effect increased with growing familiarity, a finding they explained based on inhibition theory. In our Experiment 1, the stimulus set was small and well-known (there was a rather excessive training phase) and we argue that this might have led the participants to rely more strongly on response-retrieval.

Our main question was, whether differences in task-requirements can influence whether response-retrieval is carried out. In the next experiment we therefore investigated, whether these results hold for the case of semantic comparison.

## 3. Experiment 2

As outlined before, we investigate the retrieval of the prime response in a semantic comparison task. In general, we conducted a replication of Ihrke et al.'s (2011) study subject to minor variations that make the replication comparable to Experiment 1. The differences between their study and the current one include a different set of priming conditions and a larger stimulus set. For the same reasons as outlined above, we hypothesize that there will be an interaction between response-relation and priming for the TT condition. However, we hypothesize that this interaction will also be significant for the DT and DTTD conditions, because the focus on semantic identification would cause semantic information to be matched by the retrieval process. Therefore, the matching process should “confuse” the prime and probe displays also in conditions that feature a switch of color (as opposed to Experiment 1, where the focus was on perception).

### 3.1. Methods

#### 3.1.1. Participants

24 adults (undergraduate students from the University of Göttingen, Germany: 8 male, 16 female; mean age 23.2 years, *SD* = 4.0) took part in this experiment. None had participated in Experiment 1. All participants received course credit or were paid 8 EUR (≈ 10 USD) for taking part in the study.

#### 3.1.2. Design

Effects of stimulus repetition (priming condition) and response repetition were studied within the same 4 (priming condition) × 2 (response repetition) design as in Experiment 1. Priming condition (CO, DT, TT, DTTD) and response repetition (same vs. different) were varied within subjects.

#### 3.1.3. Materials and apparatus

The procedure was identical to the one from Experiment 1, except for the reference stimulus. The reference stimulus was no longer presented as a gray pictogram next to the selection part of the display. Instead, it was presented as a word with gray letters centered below the superimposed target and distractor.

### 3.2. Results

Trials in which an error occurred as well as trials directly following an error trial (2.5 %) were not further analyzed. Trials with response latencies of less than 250 ms or more than two standard deviations above individual means for each participant and condition were excluded as outliers (4.3 %). Aggregated mean reaction times, standard deviations, and error percentages are reported in Table 2.

**Table 2:** **Summary of reaction times (RT) and error rates (ER) for Experiment 2**.

	**Mean reaction time/Error rates^a^^b^**				**RRE^c^**	
	**Different response**		**Same response**			
	**RT**	**ER**	**RT**	**ER**	**RT**	**ER**
Control	750.9 (66.0)	3.08 (2.7)	784.2 (60.3)	3.00 (2.7)	33.3	−0.08
DT (NP)	775.1 (66.6)	2.37 (2.6)	788.8 (71.9)	3.40 (2.2)	13.7	1.03
DTTD	771.1 (67.4)	1.19 (1.8)	786.4 (68.7)	3.51 (3.7)	15.3	2.32
TT (PP)	782.7 (75.7)	4.33 (4.4)	770.5 (86.4)	1.50 (2.3)	−12.2	−2.83
	**Priming effects^d^**					
DT (NP)	−24.2	0.71	−4.5	−0.40		
DTTD	−20.2	1.89	−2.1	−0.51		
TT (PP)	−31.8	−1.25	13.7	1.50		

a *Percentage of wrong response per condition*.

b *Standard-deviation in parentheses*.

c *Response-Repetition Effect, same-different*.

d *Difference of control and priming condition*.

**Figure 3:**
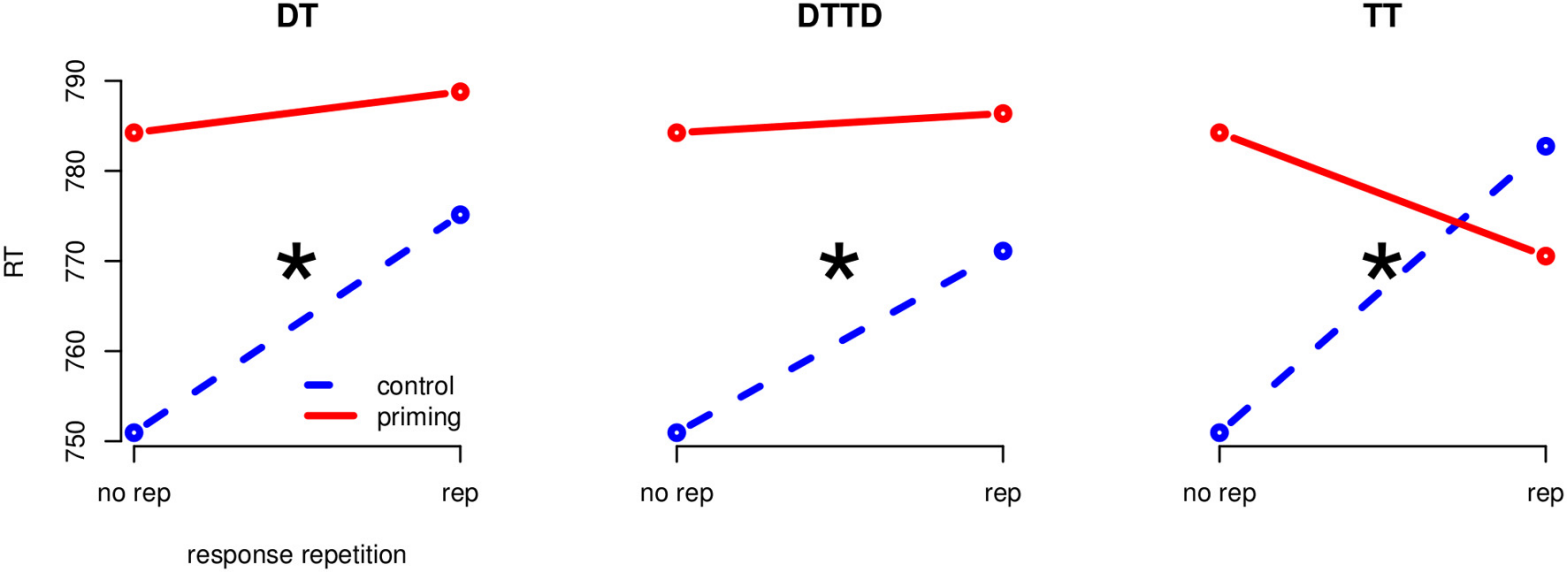
**Separate interaction plots for all priming conditions (Experiment 2)**. Blue, stippled line is the control condition, red solid line the corresponding priming condition. The “*” indicates significance at the 0.05 level.

The global 4 (priming: CO, DT, TT, DTTD) × 2 (response-repetition: same, different) ANOVA revealed a significant main effect of response-repetition, *F*_(1, 23)_ = 7.41, *MSE* = 1013.09, *p* < 0.05, η^2^_*G*_ = 0.004 and a significant priming × response-repetition interaction, *F*_(3, 69)_ = 7.34, *MSE* = 572.51, *p* < 0.01, η^2^_*G*_ = 0.007. The main effect of priming was not significant. The same separate ANOVAs per priming condition as in Experiment 1 were conducted to analyze the effects of the three stimulus repetition conditions (TT, DT, and DTTD) (Figure 3).

#### 3.2.1. Target repetition (TT)

A 2 (CO vs. TT) × 2 (same vs. different) ANOVA revealed a main effect of response-repetition, *F*_(1, 23)_ = 4.27, *MSE* = 625.33, *p* < 0.05, η^2^_*G*_ = 0.003 and an interaction between priming condition and response repetition, *F*_(1, 23)_ = 13.46, *MSE* = 922.14, *p* < 0.01, η^2^_*G*_ = 0.013. The main effect of priming was not significant. Target repetition priming delayed responding by 31.79 ms when responses in prime and probe were different, *t*_(23)_ = −2.62, *p* < 0.01, *d* = 0.30 and produced no delay but a non-significant facilitation of 13.7 ms in the response repetition condition, *t*(23) = 1.15.

#### 3.2.2. Distractor to target (DT)

A 2 (CO vs. DT) × 2 (same vs. different) ANOVA revealed a main effect of the priming condition, *F*_(1, 23)_ = 10.91, *MSE* = 454.08, *p* < 0.01, η^2^_*G*_ = 0.006, and a main effect of the response repetition, *F*_(1, 23)_ = 14.97, *MSE* = 883.31, *p* < 0.01, η^2^_*G*_ = 0.015. These effects were qualified by an interaction of priming condition by response repetition, *F*_(1, 23)_ = 9.34, *MSE* = 247.77, *p* < 0.01, η^2^_*G*_ = 0.003. Distractor to target priming had a delaying effect of 24.2 ms when responses in prime and probe were different, *t*_(23)_ = −3.92, *p* < 0.01, *d* = 0.26. In the response repetition condition no priming effects occurred, *t*(23) = −0.42, ns.

#### 3.2.3. Distractor to target and target to distractor (DTTD)

The 2 (CO vs. DTTD) × 2 (same vs. different) ANOVA revealed a main effect of response repetition, *F*_(1, 23)_ = 15.70, *MSE* = 901.17, *p* < 0.01, η^2^_*G*_ = 0.017 and of priming, *F*_(1, 23)_ = 5.65, *MSE* = 527.49, *p* < 0.05, η^2^_*G*_ = 0.004. The interaction was significant, *F*_(1, 23)_ = 9.01, *MSE* = 216.52, *p* < 0.01, η^2^_*G*_ = 0.002. DTTD priming slowed down responding by 20.16 ms when the response had to be switched, *t*_(23)_ = 03.43, *p* < 0.01, *d* = 0.23, but did not impair responding when the response was repeated, *t*(23) = −0.41, ns.

#### 3.2.4. Comparison of experiments 1 and 2

For comparing the priming × response-repetition pattern across the two experiments, we conducted separate 2 (Experiments: 1 vs. 2) × 2 response-repetition (same vs. different) ANOVAs for each priming condition, using the priming-effect (CO - priming) as dependent variable. Factor “experiment” was between-subjects, the other factors where within-subject. The relevant interaction appears as the experiment × response-repetition interaction in this analysis. This interaction was significant in the DT analysis, *F*_(1, 46)_ = 4.35, *MSE* = 1695.84, *p* < 0.05, η^2^_*G*_ = 0.030 but not in the TT, *F*_(1,46)_ = 0.26, *MSE* = 1191.35, *p* = 0.061 and the DTTD condition, *F*_(1,46)_ = 1.84, *MSE* = 390.73, *p* = 0.18. In addition, a main effect of response-repetition was significant in all analyses (DT: *F*_(1, 46)_ = 7.77, *MSE* = 1695.84, *p* < 0.01, η^2^_*G*_ = 0.053; TT: *F*_(1, 46)_ = 35.33, *MSE* = 313.28, *p* < 0.01, η^2^_*G*_ = 0.123; DTTD: *F*_(1, 46)_ = 9.68, *MSE* = 719.71, *p* < 0.01, η^2^_*G*_ = 0.066) which indicates that priming and response-repetition interacted when averaged across experiments in all priming conditions. All other effects were not significant.

#### 3.2.5. Error rates

The corresponding analyses for the error rates are largely consistent with the results from the analyses of the reaction times: The global priming × response-repetition ANOVA revealed a significant interaction, *F*_(3, 69)_ = 6.93, *MSE* = 8.34, *p* < 0.01, η^2^_*G*_ = 0.100 and no main effects. The 2 (CO vs. TT) × 2 (response-repetition) analysis revealed a main effect of response-repetition, *F*_(1, 23)_ = 4.93, *MSE* = 10.35, *p* < 0.05, η^2^_*G*_ = 0.054 and an interaction, *F*_(1, 23)_ = 5.13, *MSE* = 8.85, *p* < 0.05, η^2^_*G*_ = 0.048. In the corresponding ANOVAs for DT and DTTD, no effects reached significance.

### 3.3. Discussion

Our results were in accordance with our hypothesis and with the data by Ihrke et al. (2011): The priming by response-relation interaction did turn out to be significant over all priming conditions. Reaction times were delayed compared to control whenever stimuli were repeated (DT, DTTD, TT) but responses differed in prime and probe. For trials with ignored stimulus repetition and a response repetition between the prime and the probe no NP specific delay was observed, while a facilitative effect was noted in the TT condition. These findings support the assumptions of Rothermund et al. (2005) who predicted a dependency of priming effects on response repetition.

The results can be explained by the interpretation proposed above: Since the subject's focus was on semantic rather than perceptual identity, the memory trace created during prime processing should contain less perceptual and more semantic features and/or the perceptual scanning process should be more sensitive to the semantic similarity between the repeating stimuli. This led to the observed interaction: a delay in the case of a mismatch with the currently required response and no effect in the case of a match. Obviously, a facilitative effect for response-matches in the DT/DTTD conditions would seem to be more consistent with this argument. However, the facilitation by the retrieval of the response could have been canceled out by a less efficient matching of the scanning process due to the differences between the displays, or a less efficient coding of the distractor in the prime memory trace.

The response-repetition effect was significant over all priming conditions but was qualified by a priming × response-repetition interaction. The RRE was manifest as facilitation in the TT and delay in all other conditions whenever the response had to be repeated. Marczinski et al. (2003) suggested that a response-repetition in conjunction with stimuli that are classified as new will lead to an increase in reaction time, because the response is bound to a different stimulus. In control trials the stimuli are clearly new and correspondingly, the RRE is delaying. In TT trials, the stimuli are clearly repeated and therefore the RRE is facilitative and in the partial repetition-trials where only some features are repeated (DT and DTTD), the effect was still delaying but significantly different from the control trials. Therefore, even though the delaying effect in DT and DTTD seems to contradict the hypothesis outlined above that the orienting process recognized the trial with repeated stimuli as similar, the significant difference between control and partial repetition trials supports the conclusion that the stimuli were not classified as new either.

## 4. General discussion

The aim of the present paper was to examine the impact of the repetition of different features and of different task requirements during encoding of the prime memory trace and the retrieval of the prime response in the probe. We could confirm that retrieval depends on the task by conducting two 2-AFC NP experiments that manipulated the features that reappeared and in the depth of processing required for correct responding. While Experiment 1 realized a perceptual comparison task, the comparison had to be raised to the semantic level in Experiment 2. The results show that response-retrieval does not play a major role in the perceptual comparison task, while it is the determining process present in the semantic comparison task. We argue that it is necessary to augment the response-retrieval framework by a perceptual scanning process as already proposed by Milliken et al. (1998): The flexibility of this process together with the response-retrieval idea form a theoretical framework that can consistently explain our findings.

In the perceptual matching task (Experiment 1) an interaction between the stimulus repetition and the response repetition was only detectable for stimuli that were perceptually identical in prime and probe (TT trials). When the repeated stimuli differed with respect to the perceptual feature color, the relationship disappeared. This shows that stimulus-similarity for only some of the features (shape) in the prime was not sufficient to invoke response retrieval in the probe but that all object features had to be matched (color and shape). Nevertheless, the repetition of previously ignored stimuli in the DT and DTTD-trials was noted by the cognitive system: Delayed reaction times were observed for these conditions as compared to control – a NP effect. Although this reappearance had a measurable delaying effect on reaction times, the delay was obviously not caused by the retrieval of the prime response; otherwise, the interrelationship between the stimulus repetition condition and the response repetition would have been observed. In contrast, in Experiment 2 an interrelationship between stimulus- and response repetition was detected not only for target repetitions (TT), but also in the conditions with only partial repetition (DT and DTTD). There was no main effect of priming and therefore response-retrieval seems to be the dominating factor when semantic comparisons are used. The cross-experiment analysis confirmed this conclusion: While the factor “experiment” did not have an impact on the stimulus- × response-repetition interaction in the TT condition, the interaction was modulated significantly by experiment in the DT condition. The same analysis for the DTTD condition failed to become significant. We attribute this finding to the rather strong perceptual similarity in DTTD trials (both stimuli are repeated). Similar to the TT condition, the perceptual similarity could have resulted in a faster detection of the mismatch such that the size of the effect was too small to be detected in the global analysis (though the stimulus- × response-repetition interaction did turn out significant in the analysis restricted to Experiment 2).

We argue that this rather complex pattern of results is best explained by the temporal discrimination theory (Milliken et al., 1998) in conjunction with the response-retrieval account (Rothermund et al., 2005). The temporal discrimination theory postulates a perceptual scanning process that is initiated early during trial processing. Its objective is to estimate the likelihood that the retrieval of previously stored content in episodic memory can help to resolve the current task. This idea has its root in Logan's (1988) instance theory of automation which suggests that two competing processes are at work during responding. While slow, algorithmic processing is necessary for new, and yet unknown stimuli, in the case of repeating situations it is often helpful to predict the required response by the response that has been performed during the earlier encounter.

Our results fit nicely with this idea if we assume that the perceptual scanning process eventually decides about whether or not the prime response is retrieved (or, equivalently, whether the automatically retrieved response is used as a prediction for the current response): In the case of a perceptual comparison task, the focus during trial processing is on perceptual features rather than semantics because superficial processing was sufficient for successful performance. Therefore, these features are prominently stored in memory and readily accessed by the scanning process which could therefore determine a mismatch in the case of dissimilarities preventing an automated retrieval of the response. In the semantic comparison task, the semantic identity instead of perceptual features were relevant for responding. Therefore, the semantic identity was encoded, putting less strength on perceptual similarity and the scanning process would match semantically identical objects that differed in perceptual features. The resulting retrieval of the response-information produced the observed stimulus- × response-repetition interaction. With the same argument, the interaction between positive priming (TT) and response-retrieval present in both experiments is naturally explained: The identical repetition of the target provides strong evidence for the suitability of response-retrieval to improve performance and response-retrieval is therefore initiated in both a perceptual and a semantic comparison task.

Investigation of the response-repetition effects (RRE) in our data supports our previous conclusion. Reaction times were delayed whenever the prime response had to be repeated, but perceptual aspects of a display were “new”. Such a response repetition effect is expected as long as a response has to be repeated in cases where the presented stimuli differ (Marczinski et al., 2003). Only prime and probe displays for the control condition differ in this manner: In the TT-, DT- and DTTD-trials previously processed stimuli reappear in the probe display. However, in spite of this reappearance, the repetition of the prime response in the DT- and DTTD-trials was as improbable as in the control trials in the perceptual comparison task while it was significantly different when semantic comparison was required. This provides further evidence for the notion that no retrieval of the prime response was carried out in these conditions because the perceptual scanning process was unable to determine the similarity between prime and probe when object color was changed.

The two experiments reported in this study differed in the arrangement of the stimuli (see Figure 1): While the perceptual comparison task (Experiment 1) implemented a side-by-side layout, the reference word in the semantic comparison task was presented below the target and distractor stimuli. This setup was chosen for comparability with the previous experiments, i.e., with (Ihrke et al., 2011) for semantic and with the study by Kramer and Strayer (2001) for perceptual comparison. In the light of recent results showing that perceptual grouping can have an effect of what is encoded in an event-file (Frings and Rothermund, 2011), one might ask whether this different perceptual arrangement could have caused the observed differences in the pattern of results of the two experiments. However, this explanation can be ruled out for two reasons. First, the location of the distractor and target-stimuli were identical in both experiments, only the reference stimulus was positioned differently. Therefore, if any perceptual grouping effect was present, it would concern the reference stimulus which is of no concern for the priming effects. Second, the emergence of the stimulus- × response-repetition interaction in the control vs. TT analysis which was present in both experiments documents that stimulus-response bindings were formed under both the perceptual and the semantic comparison task. We conclude that the layout of the stimuli cannot account for our pattern of results.

One way to interpret our findings is to assume that depending on the task context, the content of the episodic memory traces would be different. In a NP context, empirical evidence for such an assumption can be derived from results obtained under the label of selective NP (Tipper et al., 1994; Frings and Wentura, 2006). These studies investigated NP for both response-relevant and irrelevant dimensions: One dimension (e.g., color) classified the role of the objects (target or distractor), and a second dimension (e.g., location) had to be responded to while a third dimension (e.g., object-identity) was completely irrelevant for the task. NP conditions can therefore be implemented in both, the completely irrelevant dimension and the classification-relevant dimension. A consistent finding is that the NP-effect is stronger when the prime distractor becomes the probe target on the relevant compared to the irrelevant dimension. Therefore, given that NP is at least partially determined by memory retrieval, the content of the memory episodes was clearly different for the relevant and irrelevant dimension.

However, our results do not necessarily imply that the memory trace or event-file created during prime processing was different in the two experiments. It is equally possible that the binding was similar but that the retrieval process itself was modulated by the requirements in the probe. Because we varied the task between experiments it was identical in prime and probe and we cannot distinguish between effects during encoding and retrieval. A logical extension of the the current study would implement a trial-wise switch of perceptual vs. semantic-matching task. This approach would provide us with the means to directly compare prime-semantic/probe-perceptual and prime-perceptual/probe-semantic trials (and vice versa) which is necessary to conclude about whether encoding or retrieval was responsible for the results reported in the current study.

The results of Experiment 1 raise another issue: Obviously negative priming in the DT and DTTD conditions was not caused by response-retrieval in this setting. Still, a global response delay was observable in the DT and the DTTD condition - a main effect of NP. While this effect can be explained also by temporal discrimination theory—a partial mismatch is harder to detect than a full mismatch—it can also be interpreted as pointing toward a second process besides retrieval that was responsible for the interference of distractor-to-target repetitions, e.g., inhibitory processes (e.g., Tipper, 1985; Tipper and Cranston, 1985). Inhibition theory assumes that an abstract, cognitive representation of the distractor object is inhibited below baseline and that it therefore requires more time to activate this representation once it is attended in the probe (resulting in the NP effect). Inhibition and response-retrieval could have been active at the same time such that the effects of both processes would overlap. Evidence for the simultaneous activity of inhibitory and retrieval processes has been found in a recent study where stimulus-response bindings were prohibited by a spatio-temporal separation of stimulus and response (Ihrke et al., 2013): When response-retrieval was removed as a possible explanation of NP, the signature priming× response-repetition interaction disappeared leaving a main effect of priming. This line of reasoning is also congruent with the results from Experiment 2: DT and DTTD were clearly delayed in the different-response condition but not facilitated in the same-response condition (as would be expected by pure response-retrieval). If response-retrieval and inhibition were at work simultaneously and largely independently, this could produce just that pattern. If, in the absence of response-retrieval, an inhibitory process produces a global response-delay (Experiment 1), the superposition of inhibition and response-retrieval would result in a stronger NP effect for response-switches and a disappearing NP effect for the response-repetition condition. Indeed, that is what appears to be the case when comparing Tables 1, 2 visually: Numerical values of the NP effects were larger in Experiment 2.

We attributed the difference between the DT and TT conditions with respect to the stimulus- × response-repetition interaction in Experiment 1 to the difference in perceptual similarity of the repeated object. While this interpretation appears to be plausible especially in the light of the results of Experiment 2 which lacked this difference between the DT and TT conditions, it could in principle also have been caused by attention rather than similarity. In the TT condition, the repeated stimulus is twice attended while it is attended only in the prime in the DT condition. A possible experiment to decide between the two interpretations could implement a trial-wise switch in target-color. This approach would allow us to compare target-target repetitions with and without perceptual similarity which would be necessary to resolve this issue. In addition, it would be beneficial to include a full repetition condition of both target and distractor (DDTT) to further disentangle if extreme perceptual similarity can increase the observed effect.

In summary, our results stress the importance of regarding response repetitions and response switches in relation to the repetitions of attended and non-attended stimuli as a factor for performance in selective attention tasks, especially with respect to negative priming. The response-retrieval theory (Rothermund et al., 2005) proposed that incidental stimulus response association was the underlying mechanism producing NP initiated in the case of stimulus repetitions. The present data show that the likelihood of response-retrieval in the case of stimulus-repetitions is affected by the encoding and retrieval of the prime memory trace. A candidate mechanism that can explain this effect is the perceptual scanning process proposed by Milliken et al. (1998): Retrieval is initiated if repeating features are encountered, but is suppressed if no repetition of features is detected.

### Conflict of interest statement

The authors declare that the research was conducted in the absence of any commercial or financial relationships that could be construed as a potential conflict of interest.

## Footnotes

^1^The reason for including these trials was that data from the current experiment is planned to be compared with a group of old adults. Differences between ST and CO trials can be used to quantify the baseline impact of distracting information and can differ between age groups (Titz et al., 2008).

^2^The reported effect size is Generalized Eta-Square, η^2^_*G*_ (Bakeman, 2005).
